# Frequency-Specific Abnormalities of Intrinsic Functional Connectivity Strength among Patients with Amyotrophic Lateral Sclerosis: A Resting-State fMRI Study

**DOI:** 10.3389/fnagi.2017.00351

**Published:** 2017-11-07

**Authors:** Fangjun Li, Fuqing Zhou, Muhua Huang, Honghan Gong, Renshi Xu

**Affiliations:** ^1^Department of Neurology, The First Affiliated Hospital of Nanchang University, Nanchang, China; ^2^Department of Radiology, The First Affiliated Hospital of Nanchang University, Nanchang, China; ^3^Jiangxi Province Medical Imaging Research Institute, Nanchang, China

**Keywords:** amyotrophic lateral sclerosis, fMRI, intrinsic functional connectivity strength, brain, cortex

## Abstract

The classical concept that amyotrophic lateral sclerosis (ALS) is a degenerative disorder characterized by the loss of upper and lower motor neurons is agreed. However, more and more studies have suggested the involvement of some extra-motor regions. The aim of this study is to investigate the frequency-related alteration pattern of intrinsic functional connectivity strength (FCS) at the voxel-wise level in the relatively early-stage of ALS on a whole brain scale. In this study, 21 patients with ALS and 21 well-matched healthy control subjects were enrolled to examine the intrinsic FCS in the different frequencies (slow-4: 0.027–0.073 Hz; slow-5: 0.01–0.027 Hz, and typical band: 0.01–0.1 Hz). Compared with the control subjects, the ALS patients showed a significantly decreased FCS in the left prefrontal cortex (PFC) and the bilateral superior frontal gyrus. In the slow-5 band, the patients with ALS showed decreased FCS in the left lingual gyrus, as well as increased FCS in the left postcentral gyrus/paracentral lobule (PoCG/PARC). In the slow-4 band, the ALS patients presented decreased FCS in the left and right ventrolateral PFC. Moreover, the increased FCS in the left PoCG/PARC in the slow-5 band was positively correlated with the ALSFRS-r score (*P* = 0.015). Our results demonstrated that the FCS changes in ALS were wide spread and frequency dependent. These findings may provide some evidences that ALS patients have the consistent impairment in some extra-motor regions at a relatively early-stage.

## Introduction

Amyotrophic lateral sclerosis (ALS) is a degenerative disorder selectively affecting motor neurons. A recent investigation suggested that ALS might involve some extra-motor neuronal networks (van der Graaff et al., 2009). Currently, advanced neuroimaging techniques provide an important tool to study ALS-related cortical function and reorganization, including the motor (Tessitore et al., 2006; Agosta et al., 2011; Zhou et al., 2013; Zhou F. et al., 2014) and the extra-motor (van der Graaff et al., 2009; Crespi et al., 2014; Zhou et al., 2016) networks. In the motor network, ALS patients show task-related dysfunctions in the motor area (Stanton et al., 2007; Mohammadi et al., 2011), decreased (Jelsone-Swain et al., 2010) and/or increased (Agosta et al., 2011) alteration of the intrinsic functional connectivity (iFC) (Zhou et al., 2013; Zhou F. et al., 2014), motor cortex atrophy (Roccatagliata et al., 2009; Schuster et al., 2014; Zhang et al., 2014), as well as the motor-related structural fiber abnormalities involving disruption of the corticospinal tracts (Zhang et al., 2014; Steinbach et al., 2015) and selective impairment of callosal fibers connecting motor and premotor cortices of the two sides (Kim et al., 2014). While motor network degeneration is the pathogenic hallmark of the disease, increasing evidence supports a widespread extra-motor neurodegenerative involvement (Hartung et al., 2014), predominantly within the frontal lobes (Roccatagliata et al., 2009; Schuster et al., 2014) and hippocampus (Steinbach et al., 2015).

Recently, the graph theory-based network analysis was applied to characterize iFC within the whole-brain functional connectome, providing more functional architecture information for ALS (Zhou et al., 2016). The functional connectivity strength (FCS), the ratio of the measured connections and the theoretical connections for a given voxel, are the class index of connectome analysis, allowing us to assess the functional importance degree in the connectome. Differently from the aforementioned functional studies, based on networkwise functional connectivity analysis through the independent component analysis or the seed-based correlation analysis, this graph-based FCS measure could characterize the functional relationships of a given node (voxel) within the entire connectivity matrix of full-brain functional connectome rather than represent the relationships with specific nodes or networks (Zuo et al., 2012). In addition, the recent study has demonstrated that the neuronal intrinsic activity is sensitive to the specific frequency bands in the amplitude of the oscillatory level (Zuo et al., 2010), as well as iFC in the regional (Song et al., 2014), interregional (Zhou F. et al., 2015), and network levels (Xue et al., 2014). The connectivity of specific frequency bands might provide more useful information. For example, the study of Iyer et al. (2015) found that frequency (alpha band) increased in the cross-spectral density of electroencephalography (EEG) in patients with ALS. Although it is not completely clear from the text the meaning and relevance of the oscillatory frequencies explored in the study (0.01–0.1 Hz) and their relationship with the EEG oscillation ranges (from theta-delta to gamma range), their consistencies are higher. Ma et al. (2016) showed that the alteration of fractional amplitude of the low-frequency fluctuations in ALS were wide spread and frequency dependent. It would be noted that the brain rhythms of the intrinsic activity represented an organized architecture of brain function and might help to get a clearer understanding of the pathophysiological mechanisms of information processing in patients with ALS.

Based on our previous works, we hypothesized that the altered intrinsic FCS in the different low-frequency bands were different in patients with ALS and that these alterations on the specific bands were correlated with the clinical manifestations of ALS. According to that, we examined the changes of the intrinsic FCS using the different frequencies (slow-4: 0.027–0.073 Hz; slow-5: 0.01–0.027 Hz, and typical band: 0.01–0.1 Hz) between ALS patients and health control subjects (HCs).

## Materials and Methods

### Participants

All subjects provided their written informed consent to participate in this study. The present study was approved by the local ethics committee and the institutional review board of the First Affiliated Hospital of Nanchang University.

Twenty-three right-handed sporadic definite ALS patients (7 women and 16 men; mean age 52.86 ± 9.45 years) and 23 right-handed age- and sex-matched HCs were recruited from the Department of Neurology at the First Affiliated Hospital of Nanchang University and the local community, respectively. All patients had a definitive diagnosis of ALS according to the revised El Escorial criteria (Brooks et al., 2000), with the clinical evidences of both upper and lower motor neuron involvement. The exclusion criteria were as follows: (a) a family history of motor neuron diseases; (b) the clinical diagnosis of frontotemporal dementia; (c) a history of brain injury, epilepsy, or other related neurologic disease; and (d) psychiatric disorder. The mean disease duration from disease onset to the date of magnetic resonance imaging (MRI) examination was 16.24 ± 10.65 months. The ALS Functional Rating Scale-revised (ALSFRS-r) was used to assess disease severity. The mean score was 33.67 ± 5.00, reflecting an early-stage of disease (lower ALSFRS-r indicates greater severity). The rate of disease progression [(48–ALSFRS-r)/disease duration] (Kimura et al., 2006), a prognostic biomarker, was 1.48 (0.25–5.34), predicting a relatively short survival time. The control subjects were screened with a regular neurological examination and presented no history of neurological or psychiatric disorders. All MRI scans were visually inspected by a radiologist to rule out a major neuropathology, such as tumor, stroke, or advanced white matter disease.

### MRI Data Acquisition

All patients and control subjects were scanned using a Trio 3.0-tesla scanner system (Siemens Medical Solutions, Erlangen, Germany). Subjects were required to keep their eyes closed, stay awake, and keep their mind as free of thoughts as possible. (1) The parameters of the resting state functional MRI (rs-fMRI) scan were as follows: repetition time/the echo time = 2000/30 ms, field of view = 220 mm × 220 mm, matrix = 64 × 64, and interleaved axial slices = 30 at a thickness of 4 mm with an interslice gap of 1.2 mm. This acquisition sequence generated 240 volumes in 8 min. (2) A high-resolution and three-dimensional (3D) T_1_-weighted sequence was established for anatomical images: The repetition time/the echo time = 1900 ms/2.26 ms, matrix = 240 × 256, field of view = 215 mm × 230 mm, sagittal slices = 176 at a thickness of 1.0 mm with no gap. (3) A conventional T_2_-weighted and fluid-attenuated inversion recovery imaging protocol was used to exclusively diagnose the patients. At the end of the scanning sessions, the subjects confirmed that they had not the fallen asleep with an Epworth sleepiness scale questionnaire.

### Functional Data Preprocessing

The preprocessing of rs-fMRI images was performed using a toolbox for Data Processing and Analysis of Brain Imaging^1^ (Yan and Zang, 2010) based on the statistical parametric mapping (SPM8^2^), which was run on Matlab 8.4.0 (Mathworks, Natick, MA, United States). This preprocessing was performed with the following steps: (1) the first 10 volumes from each subject were discarded to allow for the signal stabilization due to eliminating the magnetic saturation effects and the adaptation of subjects to the circumstance; (2) the remaining 230 volumes were corrected for the slice timing and the voxel-specific head motion calculations and corrections to adjust the time series of the images (head motion was <2 mm of translation along any axis and <2.0° of angular rotation along any axis); based on these criteria, two ALS patients and 2 well-matched healthy controls were excluded from our analyses; (3) there were no significant differences in the head motion between the ALS patients (*n* = 21) and the HCs (*n* = 21) according to the frame-wise displacement (FD) criteria described by Van Dijk et al. (2012) (0.593 ± 0.044 vs. 0.433 ± 0.022, two-sample *t-*tests, *t* = 1.488, *P* = 0.147); (4) the high-resolution individual T_1_-weighted images were co-registered to the mean functional image after the motion correction using a linear transformation; (5) the co-registered functional images were normalized to the Montreal Neurological Institute (MNI) space with 3 mm × 3 mm × 3 mm re-sampling using the transformation information acquired from the T_1_-weighted images segment with Diffeomorphic Anatomical Registration Through Exponentiated Lie; (6) the nuisance linear regression was performed with the white matter, cerebrospinal fluid, global signal, six head motion parameters, 6 head motion parameters at one time point earlier, and 12 corresponding squared items (Friston 24-parameter model) as covariates.

### Functional Connectivity Strength Mapping and Analysis

Based on our previous works (Han et al., 2011; Gao et al., 2015; Zhang et al., 2015), this study utilized FCS to examine the regional intrinsic neural activity at the different frequency bands in ALS patients. The exact frequency bins that were used in this study mainly focused on slow-5 and slow-4 because these frequency bins belonged to the typical low-frequency bands, which were considered to primarily contribute physiological manifestations in the blood-oxygen level dependent fluctuation analyses. To obtain each subject’s whole-brain FCS at the voxel level (*N*_voxels_ = 271633 in whole brain), Pearson’s correlations were first calculated between the time courses of each voxel (*x*_i_*x*) and other voxel (*x*_j_*x*) at the typical frequency bands (0.01–0.1 Hz), the slow-4 (0.027–0.073 Hz) and slow-5 (0.01–0.027 Hz) bands, which, respectively, generated a correlation matrix of the whole brain. For each *x*_i_*x*, if the functional connectivity was significant (*r*_0_*r* > 0.25) (Zuo et al., 2012; Yan et al., 2013; Zhou F.Q. et al., 2015), it was counted as an actual functional connection to *x*_0_. In the present study, the FCS was the ratio of the measured connections and the theoretical connections $\left(\frac{n(n-1)}{2}\right)$ for a given voxel, in Equation (1):

(1) $$\begin{array}{c} FCS(i)=\frac{2}{n(n-1)}\sum_{j\;=\;1}^{N}r_{ij}(r_{ij}>r_{0}) \end{array}$$

The regions with the high FCS play the important roles in the brain networks (namely hubs) (Buckner et al., 2009; Yan et al., 2013; Hou et al., 2014). Finally, by subtracting the mean FCS across the entire brain and then dividing by the standard deviation of the whole-brain FCS, these individual-level voxel-wise FCS maps were then standardized to *z*-scores (Buckner et al., 2009). A smoothing kernel of 6-mm full-width-half-maximum isotropic Gaussian kernel was applied for the statistical analysis.

### Statistical Analysis

To investigate the interactions between the disease and frequency (slow-5 and slow-4) bands, we performed a two-way analysis of variance (ANOVA) (double-factor, 2 × 2) using the SPM8 software program (Wellcome Institute of Cognitive Neurology, London, United Kingdom) voxel by voxel, and gender, age, and the mean FD were covariates (Han et al., 2011; Gao et al., 2015; Zhang et al., 2015). The further *post hoc* two-sample *t*-tests were performed for the group comparison of the slow-5 band and slow-4 band results. All significance tests were conducted based on Gaussian random field (GRF) theory with a voxel level of *P* < 0.01 and a cluster level of *P* < 0.05. A further correlation analysis between the FCS value for the significantly different brain areas and the clinical evaluations performance was then performed on the ALS groups by extracting the significantly different frequencies between two groups. A partial correlation analysis was performed to examine the relationships between the abnormal FCS and the standardized clinical evaluations scores using the SPSS 13.0 software program (SPSS, Inc., Chicago, IL, United States).

## Results

### FCS Analyses in Typical Frequency Band (0.01–0.1 Hz)

**FIGURE 1:**
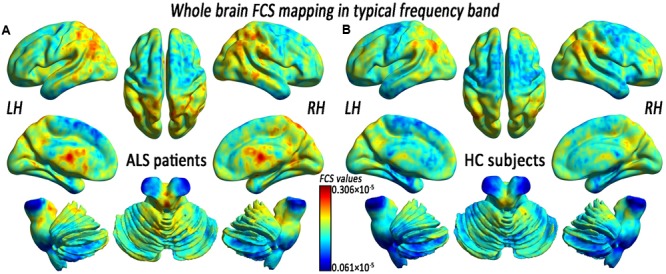
The whole-brain functional connectivity strength (FCS) patterns in the amyotrophic lateral sclerosis (ALS) patients and the healthy control subjects (HCs) in the typical frequency band. **(A)** The whole-brain FCS patterns in ALS. **(B)** The whole-brain FCS patterns in HCs. The cool color indicates the brain regions of the decreased FCS, the warm color indicates the brain regions of the increased FCS on the comparison between the ALS and HCs groups. There was no significant difference in the mean whole-brain FCS patterns in the typical frequency band between the ALS and the HCs groups. LH, left hemisphere; RH, right hemisphere.

Before comparing the FCS significances between two groups, we first reported the FCS results from the typical frequency band (0.01–0.1 Hz) separately. Both the ALS patients and the HCs showed a significantly higher FCS value level than the global average in some regions, including the posterior cingulate cortex/precuneus, the bilateral ventral medial prefrontal cortex (PFC) and the dorsolateral PFC, the bilateral middle temporal gyrus (MTG), the bilateral thalamus, and the visual cortex (**Figure 1**). We noted that many highly FCS regions were the components of what is known as the most prominent intrinsically connected hubs, namely the default mode network. The distributed alteration of FCS was observed in the ALS group, but with no statistically significant difference in the mean FCS (0.172 × 10^-5^ ± 0.080 × 10^-5^ vs. 0.156 × 10^-5^ ± 0.053 × 10^-5^) compared with the whole brain (**Table 1**).

**Table 1:** Significant differences in functional connectivity strength (FCS) between amyotrophic lateral sclerosis (ALS) and health control subjects (HCs) groups in typical frequency band (0.01–0.1 Hz).

Brain regions (ALS < HCs)	BA	Peak *T*-scores	MNI coordinates			Cluster size (voxels)	
			*x*	*y*	*z*		
Left prefrontal cortex	10,46	–4.092	–33	33	27	213
Bilateral superior frontal gyrus	8	–4.299	12	39	42	201

*ALS, amyotrophic lateral sclerosis; BA, Brodmann’s area; FCS, functional connectivity strength; MNI, Montreal Neurological Institute; the same abbreviations are used for all figures and tables.*

**FIGURE 2:**
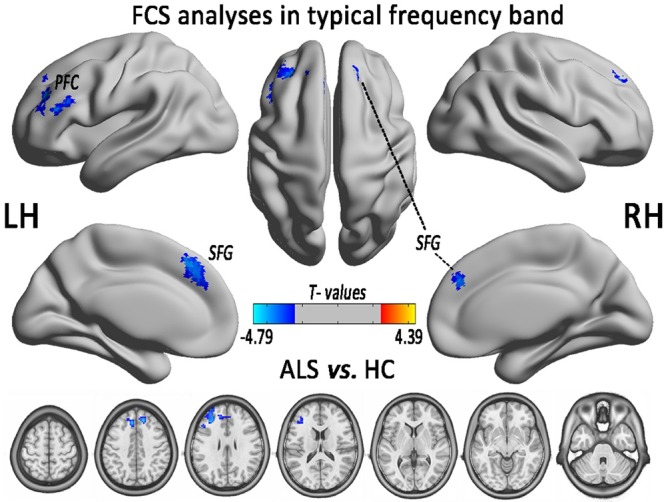
The regions of significantly decreased FCS between ALS and HCs groups in the typical frequency band (0.01–0.1 Hz). The effects are significant at the voxel level of *P* < 0.01 and the cluster level of *P* < 0.05 of GRF correction, after controlling for age and sex. The cool color indicates that the ALS group had a significantly decreased FCS compared with the HCs.

In the comparison of voxel-level FCS, the regional decreases exhibited that the ALS patients had lower FCS values than did the HCs in the left PFC (PFC) and the bilateral superior frontal gyrus (SFG), after controlling for age and sex (GRF correction, the voxel level *P* < 0.01 and the cluster level *P* < 0.05; **Figure 2** and **Table 1**). No region with the increased FCS was found in the ALS group.

### FCS Changes in Slow-5 and Slow-4 Frequency Bands

**FIGURE 3:**
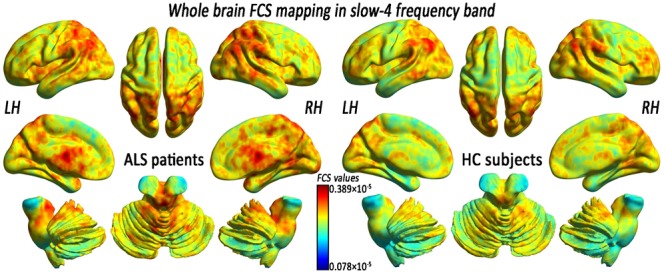
The whole-brain FCS pattern in the ALS patients and the HCs in the slow-4 frequency band. The left and right sides showed the voxel-level mean FCS pattern in the slow-4 frequency band in the ALS patients and the HCs, respectively. There was no difference in the mean whole-brain FCS pattern in the slow-4 frequency band between the ALS and the HCs groups.

**FIGURE 4:**
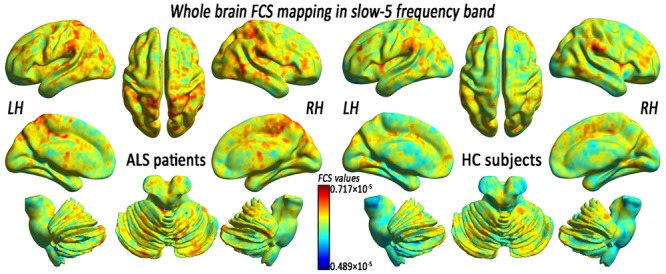
The whole-brain FCS pattern in the ALS patients and the HCs in the slow-5 frequency band. The left and right sides showed the voxel-level mean FCS pattern in the slow-5 frequency band in the ALS patients and the HCs, respectively. There was no difference in the mean whole-brain FCS pattern in the slow-5 frequency bands between the ALS and the HCs groups.

**FIGURE 5:**
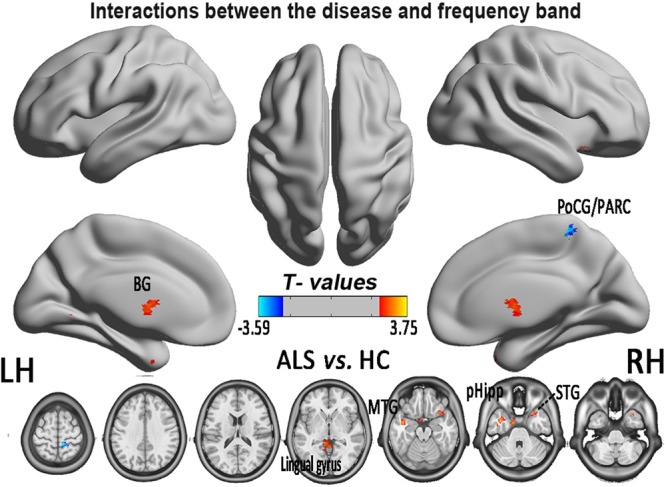
The interaction between the frequency band and the diseased FCS. The alterations indicated the interaction between the frequency bands (slow-4 and slow-5) and the groups of ALS patients and HCs, based on the two-way ANOVA (double-factor), with Gaussian random field correction, the voxel level of *P* < 0.05 and the cluster level of *P* < 0.05 were considered to be statistically significant. The cool color indicates the brain regions of the decreased FCS, and the warm color indicates the brain regions of the increased FCS on the comparison between ALS and HCs groups. CAL, cerebellum anterior lobe.

In the FCS pattern in the whole brain in the slow-4 and slow-5 frequency bands (shown in **Figures 3, 4**, respectively), no difference was detected between the patients with ALS and the HCs. However, a significant interaction was identified in the right superior temporal gyrus (STG), the left Parahippocampal gyrus (pHipp), the left MTG, the left lingual gyrus, the bilateral basal ganglia, and the right postcentral gyrus/paracentral lobule (PoCG/PARC) between the frequency bands and groups (**Figure 5** and **Table 2**).

**Table 2:** Significant interaction effects between frequency band (slow-4 and slow-5) and groups by a two-way ANOVA (double-factor) and a *post hoc* two-sample *t*-test.

Brain regions	BA	Peak *t*-scores	MNI coordinates			Cluster size
			*x*	*y*	*z*	(voxels)
**Interaction between frequency band and ALS disease**					
Right superior temporal gyrus	38	3.544	33	18	–39	49
Left Parahippocampal gyrus	28	3.206	–12	–3	–30	45
Left middle temporal gyrus	21,38	3.751	–42	–3	27	77
Left lingual gyrus	30	3.216	–3	–36	–6	98
Bilateral basal ganglia		2.909	0	0	6	32
Right PoCG/PARC	5	–3.591	12	–39	63	38
**In slow-5 band (ALS < HCs)**					
Left lingual gyrus	30,19	–3.946	–12	–45	0	80
ALS > HCs						
Left PoCG/PARC	7,6	4.411	–6	–27	66	160
**In slow-4 band (ALS < HCs)**					
Right VLPFC	10	–4.032	42	51	12	244
Left VLPFC	10	–3.572	–48	24	24	146
ALS > HCs
None

*PoCG, postcentral gyrus; PARC, paracentral lobule; VLPFC, ventrolateral prefrontal cortex. *T* statistical value of peak voxel showing FCS differences. All clusters had GRF correction at voxel level of *P* < 0.01 and cluster level of *P* < 0.05.*

**FIGURE 6:**
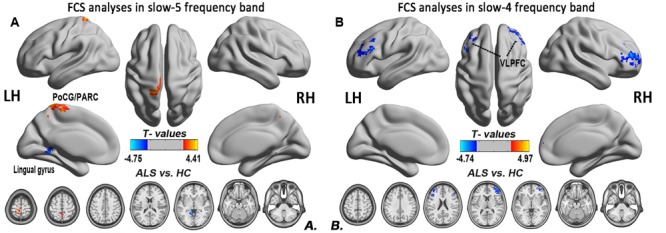
The differences in the FCS between the ALS patient and HCs groups in two low-frequency bands. **(A)** The differences in the FCS between the ALS patients and HCs in the slow-5 band. **(B)** The differences in the FCS between the ALS patients and HCs in the slow-4 band (*post hoc* two-sample *t-*test; GRF correction at the voxel level of *P* < 0.01 and the cluster level of *P* < 0.05). The cool color indicates the brain regions of the decreased FCS, and the warm color indicates the brain regions of the increased FCS on the comparison between ALS and HCs groups.

To investigate the specific alterations in the ALS patients, we also subdivided the low-frequency range into two bands as previously defined: slow-5 (0.01–0.027 Hz) and slow-4 (0.027–0.073 Hz) according to Buzsáki framework (Buzsáki and Draguhn, 2004). In the slow-5 band, compared with the HCs, the ALS patients had decreased FCS in the left lingual gyrus, whereas FCS was increased in the left PoCG/PARC (GRF correction, the voxel level *P* < 0.01 and the cluster level *P* < 0.05; **Figure 6A**). In the slow-4 band, compared with the HCs, the ALS patients had decreased FCS in the left and right ventrolateral prefrontal cortex (VLPFC), whereas no increased FCS was detected in the ALS patients (GRF correction, voxel level *P* < 0.01 and cluster level *P* < 0.05; **Figure 6B**). The differences of FCS in *t*-values and the cluster size of the ALS patients vs. the HCs in the slow-5 and slow-4 bands are listed in **Table 2**.

### Relationships between Abnormal FCS Values and Neuropsychological Assessments

**FIGURE 7:**
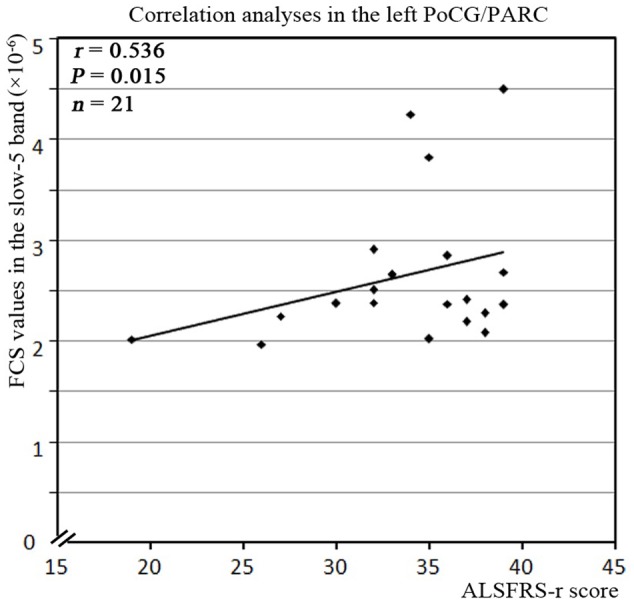
The correlation between the abnormal clusters of FCS in the slow-5 band and the neuropsychological assessments in the ALS patients. The abnormal clusters of the FCS in the slow-5 band were significantly correlated with the ALSFRS-r scores in the ALS patients (*P* < 0.05, with bootstrapping statistics). A relationship was observed between ALSFRS-r score and the increased FCS values in the slow-5 band in the left PoCG/PARC.

For the correlation analysis, the FCS value of the ALS patients was extracted from the significantly different brain areas between groups in the different frequency bands (slow-5, slow-4) and in the typical frequency band. The correlations between the abnormal FCS values and ALSFRS-r scores were calculated for the ALS group. Only the increased FCS in the left PoCG/PARC in the slow-5 band was positively correlated with the ALSFRS-r score (*P* = 0.015) (**Figure 7**).

## Discussion

We investigated the abnormalities of the intrinsic FCS in patients with ALS in the different frequency bands (slow-4 and slow-5). First, the ALS patients showed a significantly decreased FCS in several regions. A significant interaction between the frequency band and the ALS disease was also identified in the right STG, the left pHipp, the left MTG, the left lingual gyrus, the bilateral basal ganglia, and the right PoCG/PARC. Moreover, the increased FCS in the left PoCG/PARC in the slow-5 band was positively correlated with the ALSFRS-r score (*P* = 0.015). Our findings suggested that the neuronal degeneration was related to the alteration of the intrinsic FCS and provided a novel insight that the alteration of intrinsic FCS could improve the understanding of early- stage ALS.

### Decreased Intrinsic FCS in Typical Frequency Band in ALS Patients

The intrinsic FCS has been proposed to assess the degree of functional importance in the connectome of healthy individuals (Zhou Y. et al., 2014) and in pathological brains (Zhou F. et al., 2015; Zhuang et al., 2015). The neurophysiological mechanisms that underlie the distinct frequency-band properties or the specific classes of oscillations may be caused by the different neuronal origins, the differences in cytoarchitecture, or links to the specific neural processes (Penttonen, 2003; Buzsáki and Draguhn, 2004), including the input selection, plasticity, binding, and consolidation (Zuo et al., 2010). The exact mechanism, however, remains poorly understood.

In this study, the ALS patients had several decreased-FCS regions, including the left PFC and the bilateral SFG. These findings indicated that there is widespread extra-motor neurodegeneration at the early-stage of ALS. The SFG is thought to contribute to higher cognitive functions (du Boisgueheneuc et al., 2006), such as working memory and executive and spatially oriented processing. Moreover, SFG is involved in sensory-motor coordination and has been associated with long-lasting deficits in the complex motor functions (Martino et al., 2011). In our results, the decreased FCS of left PFC was primarily located in the VLPFC. Neuroimaging studies have shown that the left VLPFC region is a critical region for processing of words and sentences, for the cognitive control of memory (Badre and Wagner, 2007), as well as for motor inhibition (Mazzola-Pomietto et al., 2009). In ALS patients with cognitive impairment, alterations in the left PFC and the bilateral SFG have been reported in several studies, including cortical atrophy (Verstraete et al., 2012; Schuster et al., 2014; Zhang et al., 2014), white matter impairment (Hartung et al., 2014; Zhang et al., 2014), reduced structural connectivity (Schmidt et al., 2014; Buchanan et al., 2015), deactivation (Mohammadi et al., 2011; Li et al., 2015), and decreased iFC (Zhang et al., 2014). In ALS patients, the pathogenic process affects both the structural and functional connectivity of the brain, indicating that the degeneration of structural and functional networks in ALS is coupled with the most prominent effects in the structural connectivity (Schmidt et al., 2014). In addition, there is a relation between ALS and frontotemporal dementia in the neuroimaging and neuropathologic findings, which provide more evidences for understanding alterations in the network connectivity due to frontotemporal lobar degeneration (Woolley and Strong, 2015). Combining the previous fMRI studies of ALS, which reported the significantly decreased functional connectivity within the frontal cortex, and our results in iFCS of the typical frequency band hint at the decreased response in the motor-sensory system, the loss of motor inhibition, and cognitive impairment.

### Differences in Intrinsic FCS between Frequency Bands

#### Altered Intrinsic FCS in Slow-5 Band in ALS Patients

In this study, we observed the decreased FCS in the left lingual gyrus, as well as the increased FCS in the left PoCG/PARC. The lingual gyrus is linked to processing vision, especially related to letters. The ALS patients exhibit a decreased iFC (Zhou et al., 2016) and spontaneous low-frequency fluctuations (Luo et al., 2012) in the visual areas, including the visual processing regions. Our findings suggested that the disorder of visual process was in accordance with the previous task-and resting-related fMRI studies (Lulé et al., 2007; Luo et al., 2012; Zhou et al., 2016). Moreover, the correlation analysis showed that the increased FCS in the left PoCG/PARC in the slow-5 band was positively correlated with the ALSFRS-r score in the ALS group after eliminating the influence of age and gender, and showed that the ALS patients with higher FCS in the left PoCG/PARC had a slightly more advanced state of this illness. The increased connectivity was confirmed in the motor network, brainstem, and the ventral attention, the default mode network in ALS patients (Menke et al., 2016; Sneha et al., 2016; Schulthess et al., 2016), which altered the patterns of association in line with the literature supporting the loss of inhibitory interneurons. The increased FCS in the left PoCG/PARC probably reflects a “compensatory” response to the disorder of sensorimotor process. The enhanced regional coherence (Zhou F. et al., 2014) and the increased iFC (Zhou et al., 2016) in the postcentral gyrus have been identified in previous studies.

#### Altered Intrinsic FCS in Slow-4 Band in ALS Patients

In the slow-4 band, ALS patients were also observed to have decreased FCS in the left and right VLPFC. In this study, the decreased FCS in the VLPFC has been observed in the typical frequency band, which probably reflected a decline in the language cognitive area or the motor inhibition area. The decreasing FCS in the left VLPFC might be used to assess the severity of ALS.

### Frequency-Specific Changes in Intrinsic FCS in ALS Patients

In an fMRI study, the amplitude of low-frequency fluctuation is divided into five frequency bands, including slow-6 (0–0.01 Hz), slow-5 (0.01–0.027 Hz), slow-4 (0.027–0.073 Hz), slow-3 (0.073–0.198 Hz), and slow-2 (0.198–0.25 Hz) (Zuo et al., 2010). It has been indicated that the differential neurophysiological manifestations that underlie the distinct frequencies may arise from the neuronal input selection and plasticity. For example, the dynamic oscillations of the high-frequency from the discharge of pyramidal cells in the receptive field “enslaves” the basket cells through resonance tuning, however, the exact mechanism remains poorly understood (Buzsáki and Draguhn, 2004). Previously, the different intrinsic brain activity patterns of specific frequency bands have been found in other diseases. These studies also suggested that the pattern of the intrinsic FCS was sensitive to the specific frequency bands. For example, in ALS patients, “compensatory” increased FCS in the left PoCG/PARC in the slow-5 band was found, but more clinical correlation was evidenced in the slow-4 band.

### Motor Cortex

**FIGURE 8:**
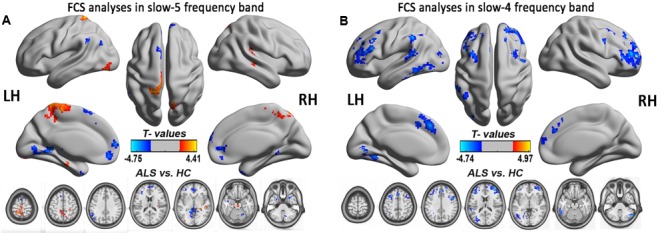
The FCS analysis in the slow-5 and slow-4 frequency bands. The significant alterations of FCS between the ALS and HCs groups in two low-frequencies of slow-4 and slow-5 bands were shown by the relatively flexible statistical threshold and the correction method (*post hoc* two-sample *t*-test, *P* < 0.05 with AlphaSim correction). **(A)** The differences in FCS between the ALS patients and the HCs in the slow-5 band (*P* < 0.05 with AlphaSim correction). **(B)** The differences in FCS between the ALS patients and the HCs in the slow-4 band (*P* < 0.05 with AlphaSim correction). The cool color indicates the brain regions of the decreased FCS, and the warm color indicates the brain regions of the increased FCS on the comparison between ALS and HCs groups.

Amyotrophic lateral sclerosis is a disorder characterized by the loss of upper and lower motor neurons, although the extra-motor areas are also involved. But in this study, finding no significant FCS difference in motor cortex was unexpected, which may be due to the statistical threshold and the correction method. To test this possibility, the relatively flexible statistical threshold and the correction methods were used for the group comparison in the slow-5 and slow-4 frequency bands. After statistics and correction, we determined that the right precentral gyrus (premotor) survived at the slow-4 frequency band (see **Table 3** and **Figure 8**). Furthermore, the right precentral gyrus had the most significantly increased FCS of the left PoCG/PARC at the slow-4 frequency band (*P* < 0.05 AlphaSim). Thus, this region survived the low threshold (*P*-value) in the AlphaSim correction, which suggested that the homogenous changes of FCS in the motor cortex occurred at the relatively early-stage of ALS.

**Table 3:** Significant alteration between ALS patients and HCs in slow-4 and slow-5 frequency bands (a *post hoc* two-sample *t*-test, *P* < 0.05 with AlphaSim correction).

Brain regions	BA	Peak *t*-scores	MNI coordinates			Cluster size	
			*x*	*y*	*z*	(voxels)	
**In slow-5 band (ALS < HCs)**							
Right cerebellum posterior lobe		–2.743	12	–66	–45	66	
Right superior temporal gyrus	38	–4.749	33	18	–39	60	
Left middle temporal gyrus	38,20	–4.023	–45	–6	–27	77	
Left medial frontal gyrus	32,10	–3.701	3	45	–6	132	
Left lingual gyrus	19	–3.945	–12	–45	0	232	
Left medial frontal gyrus	46,10	–3.398	–33	45	3	158	
Left supramarginal gyrus	40,39	–3.578	–48	–75	30	122	
Left superior frontal gyrus	6	–3.145	–9	18	57	62	
**In slow-5 band (ALS > HCs)**					
Left parahippocampal gyrus	20,35	3.836	–27	–9	–45	75	
Brain stem		3.824	–3	–24	–27	81	
Left inferior occipital gyrus	18,19	3.175	–42	–81	–15	71	
Left superior temporal gyrus	41	3.126	33	–21	0	88	
Left postcentral gyrus/paracentral lobule	5,6,7	4.411	–6	–27	66	499	
**In slow-4 band (ALS < HCs)**					
Right cerebellum posterior lobe		–4.688	51	–72	–39	74	
Left inferior temporal gyrus	37,20	–3.780	–57	–51	–21	208	
Left lingual gyrus	19	–3.5686	–24	–51	–9	115	
Left middle frontal gyrus	10,46	–4.137	–33	9	63	545
Right middle frontal gyrus	46	–4.032	42	51	12	553
Left superior temporal gyrus	22,40	–3.015	–57	–42	15	99
Left middle occipital gyrus	18,19	–3.662	–39	–69	15	74
Right middle frontal gyrus	6,8,9	–4.739	15	36	42	325
Right inferior frontal gyrus	9	–3.521	48	6	36	63
Left inferior parietal lobule	40	–4.048	–33	–51	36	113
**In slow-4 band (ALS > HCs)**					
None

### Limitations

The present study served as an exploratory study reporting the frequency-specific abnormalities of FCS in patients with ALS. There were several limitations that should be noted. First, the study had a small sample size. Second, we did not do an objective assessment of sleep quality or daytime sleepiness. The ALSFRS-r score in this study could not reflect the subtle specific cognitive alterations in ALS, which may have caused us to underestimate the multifaceted characteristics of the decreased FCS in the PFC in ALS. More sensitive and specific cognitive functioning scales should be performed in our further studies, especially those focusing on ALS cognitive deficits. Third, only ALS patients in the relatively early-stage of the disease were was included in this study, the different onset stages and the involvement of upper motor neurons, bulbar motor neurons, and spinal motor neurons at the early-stage of ALS represent the different phenotypes of the disorder and, thus, might be the reason for not detecting motor-related FCS impairment at the whole brain level and the relatively narrow correction. Finally, with regard to the reported frequency-dependent results, we mainly focused on the slow-5, slow-4, and the typical frequency bands in the ALS patients.

## Conclusion

In this study, we provided some evidence that the decreased FCS in the extra-motor regions was more consistent with the imaging feature at the relatively early-stage of ALS. Specifically, the ALSFRS-r score was related to the increased FCS of the left PoCG/PARC in the slow-5 band, which suggested that it reflected a “compensatory” response to the sensorimotor processing in the ALS patients. Furthermore, the different spatial patterns of FCS alterations in the different frequency bands also suggested that the frequency factor should be taken into consideration in exploring the ALS-related clinical manifestations or the reference of its diagnosis.

## Author Contributions

FL, FZ, and RX: Data collection, analysis, interpretation, drafting, and revising article. FZ, MH, and HG: MRI data collection. FL, FZ, and RX: Study design.

## Conflict of Interest Statement

The authors declare that the research was conducted in the absence of any commercial or financial relationships that could be construed as a potential conflict of interest.

## Footnotes

1 http://rfmri.org/dpabi

2 http://www.fil.ion.ucl.ac.uk/spm/software/spm8/
